# Interaction Network of Porcine Circovirus Type 3 and 4 Capsids with Host Proteins

**DOI:** 10.3390/v14050939

**Published:** 2022-04-29

**Authors:** Jianwei Zhou, Yongxia Wang, Linyi Zhou, Yonghui Qiu, Jie Zhao, Beining Dai, Xufei Feng, Lei Hou, Jue Liu

**Affiliations:** 1 College of Veterinary Medicine, Yangzhou University, Yangzhou 225009, China; zhoulinyi01@126.com (L.Z.); 18238776375@163.com (Y.Q.); ylsheidong@163.com (J.Z.); dbn20010622@163.com (B.D.); xffeng@yzu.edu.cn (X.F.); hlbj09@163.com (L.H.); 2 Jiangsu Co-Innovation Center for Prevention and Control of Important Animal Infectious Diseases and Zoonoses, Yangzhou University, Yangzhou 225009, China; 3 College of Animal Science and Technology, Anhui Agricultural University, Hefei 230036, China; mercy171208@163.com

**Keywords:** porcine circovirus type 3 and 4, viral capsid, protein interaction network, bioinformatics approach, GO and KEGG analyses

## Abstract

An extensive understanding of the interactions between host cellular and viral proteins provides clues for studying novel antiviral strategies. Porcine circovirus type 3 (PCV3) and type 4 (PCV4) have recently been identified as viruses that can potentially damage the swine industry. Herein, 401 putative PCV3 Cap-binding and 484 putative PCV4 Cap-binding proteins were characterized using co-immunoprecipitation and liquid chromatography-mass spectrometry. Both PCV3 and PCV4 Caps shared 278 identical interacting proteins, but some putative interacting proteins (123 for PCV3 Cap and 206 for PCV4 Cap) differed. A protein–protein interaction network was constructed, and according to gene ontology (GO) annotation and Kyoto Encyclopedia of Genes and Genomes (KEGG) database analyses, both PCV3 Cap- and PCV4 Cap-binding proteins participated mainly in ribosome biogenesis, nucleic acid binding, and ATP-dependent RNA helicase activities. Verification assays of eight putative interacting proteins indicated that nucleophosmin-1, nucleolin, DEAD-box RNA helicase 21, heterogeneous nuclear ribonucleoprotein A2/B1, YTH N6-methyladenosine RNA binding protein 1, and Y-box binding protein 1 bound directly to both PCV3 and PCV4 Caps, but ring finger protein 2 and signal transducer and activator of transcription 6 did not. Therefore, the interaction network provided helpful information to support further research into the underlying mechanisms of PCV3 and PCV4 infection.

## 1. Introduction

Porcine circoviruses (PCVs) are non-enveloped viruses containing single-stranded circular DNA genomes (~1.7–2.0 kb) within the *Circovirus* genus in the family *Circoviridae* [1]. Four genotypes of circoviruses have been detected in pigs [2,3,4]. PCV1 is non-pathogenic, whereas PCV2 is the predominant pathogen responsible for porcine circovirus-associated diseases (PCVADs) [5,6]. PCV3 was firstly found in America using metagenomic sequencing in 2015, and it is linked to different clinical symptoms, such as porcine dermatitis and nephropathy syndrome (PDNS), respiratory disease, reproductive failure, and diarrhea [7,8,9]. PCV4 was at first detected in pig farms of Hunan province, China, in 2019, and it is considered to induce serious clinical conditions, such as respiratory distress and PDNS [2]. Subsequently, PCV4 has been detected in other provinces in China [10,11,12,13], indicating that it is probably prevailing in Chinese pig farms. In addition, PCV4 has been detected in South Korea [14]. Thus, PCVs have emerged as important pathogens causing severe damage to the pig industry worldwide.

The PCV genome contains 11 predicted open reading frames (ORFs) [15,16,17]. At present, six viral proteins have been characterized. ORF1-encoded Rep and Rep’, which are replicase proteins, are required for the rolling-circle replication of the PCV genome [18,19,20]. ORF2-encoded Cap is required for virion packaging and virus propagation by binding to Rep, which is also a viral immunogen that serves as a vital controller of virus replication [21,22,23,24,25]. The PCV4 Cap consists of 228 amino acids (aa), and the PCV3 Cap contains 214 aa, while the amino acid identity between PCV3 and PCV4 Caps is below 24.5% [2]. The four other viral proteins are relevant to PCV infection but not replication [26,27,28,29]. Due to the lack of autonomous DNA polymerases, circoviruses depend on cellular replication machinery for their multiplication [30]. Thus, a comprehensive knowledge of the functions of the PCV Cap protein and its binding with host proteins would broaden our understanding of the pathogenesis of PCV infections.

As obligate parasites, viruses depend on host–pathogen protein–protein interactions to control cellular biological processes for virus propagation [31]. On the one hand, interactions between the virus and host play a crucial role in the cellular antiviral innate immune system to eliminate the invading extracellular pathogen. On the other hand, the interactions have reshaped the virus to manipulate the host defense system for its own propagation. They both all experience co-evolutionary processes [32]. Several reports have highlighted interactions between cellular proteins and PCV1 or PCV2 Cap with bacterial or yeast two-hybrid or co-immunoprecipitation assays [24,25,33,34,35,36,37,38]. However, the interactome profile of PCV3 or PCV4 Cap proteins with cellular proteins is still unclear. Furthermore, an advanced high-throughput proteomic approach has not been utilized to identify substantial PCV3 or PCV4 Cap-binding host proteins.

In the current research study, co-immunoprecipitation (Co-IP) combined with liquid chromatography-mass spectrometry (LC-MS) was employed to map the interactome profile of PCV3 or PCV4 Cap proteins. This process identified 401 and 484 putative host cellular proteins binding to PCV3 or PCV4 Cap in the transfected cells, respectively, which were used to construct a protein–protein interaction (PPI) network. Gene ontology annotation and pathway enrichment analyses demonstrated that PCV3 and PCV4 Cap-binding proteins are involved in various cellular pathways, such as RNA binding, DNA binding, ribonucleoprotein complex binding, and ATP-dependent RNA helicase activity. Eight putative interacting proteins were randomly selected for verification, and it was confirmed that both PCV3 and PCV4 Cap could interact directly with six proteins in vitro, NPM1, NCL, DDX21, hnRNPA2/B1, YTHDF1, and YBX1, albeit they exhibited varied binding capacities. Hence, the present research would be helpful in finding new antiviral therapeutic strategies against PCV3 or PCV4 infections.

## 2. Materials and Methods

### 2.1. Cells and Cell Culture

PK-15 cells were maintained in minimal essential medium (MEM) (Gibco, Carlsbad, CA, USA) supplemented with 10% fetal bovine serum (FBS) (LONSERA, Shanghai Shuangru Biology Science & Technology Co., Ltd.). HEK293T cells (CRL-11268; ATCC, Manassas, VA, USA) were cultured in Dulbecco’s modified Eagle’s medium (DMEM) (Gibco) supplemented with 10% fetal bovine serum (FBS) (Gibco) as described elsewhere [39,40].

### 2.2. Antibodies and Reagents

Mouse monoclonal antibodies (mAbs) against β-actin (M1210-2) and GST (M0807-1), and rabbit polyclonal antibodies (pAbs) against Myc (R1208-1), FLAG (0912-1), and GFP (SR48-02) were purchased from Huaan Biological Technology (Hangzhou, China). Anti-FLAG affinity resin (A2220) for immunoprecipitation was acquired from Sigma-Aldrich. Mouse anti-Myc (05-419) and anti-FLAG (F1804) mAbs were obtained from Sigma-Aldrich (St. Louis, MO, USA). NP-40 cell lysis buffer (50 mM Tris (pH 7.4), 150 mM NaCl, and 1% NP-40) was acquired from Beyotime (P0013F; Shanghai, China). Horseradish peroxidase (HRP)-labeled goat anti-mouse and anti-rabbit IgG antibodies were obtained from KPL (Milford, MA, USA). 

### 2.3. Plasmid Construction and Transfection

Full-length PCV3 and PCV4 *Cap* DNA fragments were amplified by polymerase chain reaction (PCR) from synthetic genomic DNAs of PCV3 [8] (accession no. KT869077.1) and PCV4 [2] (accession no. MK986820.1) and inserted into the multiple cloning sites of vectors pCMV-Myc-N (Clontech, Palo Alto, CA, USA) or pCMV-Flag-N (Clontech) to obtain plasmids Myc-PCV3-Cap, FLAG-PCV3-Cap, Myc-PCV4-Cap, and FLAG-PCV4-Cap. The full-length cDNA sequences of *NPM1* (accession no. XM_013990662.2), *NCL* (accession no. XM_021074959.1), *DDX21* (accession no. KX396051.1), *hnRNPA2/B1* (accession no. XM_021078978.1), *YTHDF1* (accession no. MN606020.1), *YBX1* (accession no. XM_021096922.1), *RNF2* (accession no. XM_021102630.1), and *STAT6* (accession no. HM135386.1) were amplified from PK-15 cells using specific primers, and they were subcloned separately into vector pCMV-Flag-N (Clontech). The resultant plasmids were FLAG-NPM1, FLAG-NCL, FLAG-DDX21, FLAG-hnRNPA2/B1, FLAG-YTHDF1, FLAG-YBX1, FLAG-RNF2, and FLAG-STAT6. The primers used are listed in Table 1. PK-15 or HEK293T cells were grown on plates to 70% to 90% confluency for transfection or co-transfection. The jetPRIME transfection reagent (Polyplus Transfection, New York, NY, USA) was utilized for PK-15 cell transfection, while the ExFect transfection reagent (T101-01/02; Vazyme Biotechnology, Nanjing, China) was utilized for HEK293T cell transfection, according to the manufacturer’s protocols.

### 2.4. SDS-PAGE and Western Blotting

Cell lysates extracted in NP-40 cell lysis buffer (50 mM Tris [pH 7.4], 150 mM NaCl, and 1% NP-40) after transfection or co-transfection were separated using sodium dodecyl sulfate-polyacrylamide gel electrophoresis (SDS-PAGE), transferred to nitrocellulose membranes (GE Healthcare, Chicago, IL, USA), and blocked in phosphate-buffered saline (PBS) containing 5% skimmed milk powder and 0.05% Tween 20. The membranes were then incubated with primary antibodies overnight at 4 °C, followed by incubation with the corresponding HRP-labeled secondary antibodies at room temperature for 1.0 h. The membranes were then incubated with an enhanced chemiluminescence reagent (34096; Thermo Scientific, Waltham, MA, USA), and the immunoreactive protein bands were visualized using AI800 Images (GE Healthcare).

### 2.5. Expression and Purification of Recombinant Proteins

*Escherichia coli* BL21 (pLysS) cells harboring pGEX-4T-1, pGEX-4T-1-NCL, pGEX-4T-1-hnRNPA2/B1, and pGEX-4T-1-YBX1 plasmids were cultured separately in 200 mL of Luria Bertani (LB) medium and induced with 1.0 mM isopropyl β-D-thiogalactopyranoside (IPTG) at 16 °C overnight. Cell pellets were lysed by sonication in binding buffer (1 mM PMSF, 50 mM Tris-HCl, and 150 mM NaCl pH 8.0). After centrifugation, the supernatant was incubated separately with Pierce glutathione agarose beads (21516; Thermo, Rockford, IL, USA) for 2.0 h at 4 °C and then purified. GST, GST-NCL, GST-hnRNPA2/B1, and GST-YBX1 proteins were eluted in eluting buffer containing 2.0 mg/mL of reduced glutathione, respectively.

### 2.6. Co-Immunoprecipitation (Co-IP) and Glutathione S-Transferase (GST) Pull-Down Assays

For the Co-IP assays, HEK293T cells transfected with the indicated plasmids for 48 h were lysed in NP-40 cell lysis buffer and centrifuged at 12,000× *g* for 10 min. The supernatants were treated with protein A/G plus agarose (sc-2002; Santa Cruz Biotechnology, CA, USA) for 1.0 h at 4 °C and immunoprecipitated using anti-FLAG beads. The beads were washed with NP-40 buffer and resolved using standard SDS-PAGE. For the GST pull-down assays, FLAG-PCV3-Cap or FLAG-PCV4-Cap was used as the prey protein. Equal amounts of purified GST, GST-NCL, GST-hnRNPA2/B1, and GST-YBX1 proteins, which were immobilized on glutathione agarose beads, were incubated with the corresponding prey proteins at 4 °C for 4.0 h. The precipitated proteins were washed with PBS and subjected to SDS-PAGE and Western blotting using mouse monoclonal antibodies against GST or FLAG. The Co-IP and GST pull-down assays were conducted as previously described [41].

### 2.7. Liquid Chromatography-Mass Spectrometry (LC-MS)

Coomassie blue staining gels from Co-IP experiments were mixed and subjected to protein identification via LC-MS analysis in APTBio (Shanghai, China). The peptides were concentrated and desalted on an EASY column (2 cm × 100 μm × 5 μm-C18; 75 μm × 100 mm × 3 μm-C18; Thermo Finnigan, San Jose, CA, USA) and eluted online on an analytical RP column (0.18 × 150 mm BioBasic-18, Thermo Electron, Waltham, MA, USA). A 60 min gradient was performed as follows: 4–50% B (solvent A: 0.1% *v*/*v* formic acid; solvent B: 0.1% *v*/*v* formic acid, 84% *v*/*v* ACN) 0–50 min, 50–100% B for 50–54 min, and 100% B for 54–60 min. Protein searches were performed using Mascot 2.2 software. Proteins found in the respective negative control samples were eliminated from the dataset to eliminate non-specifically bound interactions. Proteins represented by at least one unique peptide were used for further analyses. The LC-MS procedures were conducted as described elsewhere [25].

### 2.8. Construction and Analysis of a Protein-Protein Interaction Network

The experimentally derived data sets were used to plot a PCV3 and PCV4 Cap-host protein interaction network using Cytoscape software (version 3.7.1). The STRING database was used to analyze the interactions among host proteins. Only interactions confirmed via direct physical binding were considered when plotting the protein–protein interaction map. The topological parameters and central measures of the network were calculated using the network analyzer tool in Cytoscape version 3.7.1. Pig protein–protein interaction analysis was also performed using the STRING database. To reach a consensus in protein accession, we used NCBI gene names to represent proteins all throughout the study. The corresponding NCBI gene names are listed separately (Supplementary Table S2). The procedures in evaluating PPIs were performed as previously described [25].

### 2.9. GO and KEGG Pathway Analyses

Gene ontology (GO) analysis was performed using Cytoscape software (version 3.7.1) with the plugin GOclue to annotate the genes in terms of cellular component (CC), biological process (BP), and molecular function (MF) based on the GO database (Supplementary Table S4). The Kyoto Encyclopedia of Genes and Genomes (KEGG) enrichment analysis was conducted to predict the pathways based on the KEGG database (Supplementary Table S5). The GO and KEGG pathways with a corrected *p*-value < 0.05 were chosen to be significantly enriched. GO and KEGG pathway analyses were completed by APTBio (Shanghai, China) and performed as described previously [25].

## 3. Results

### 3.1. Characterization of Cap-Host Protein Interactions via Liquid Chromatography-Mass Spectrometry

**Figure 1:**
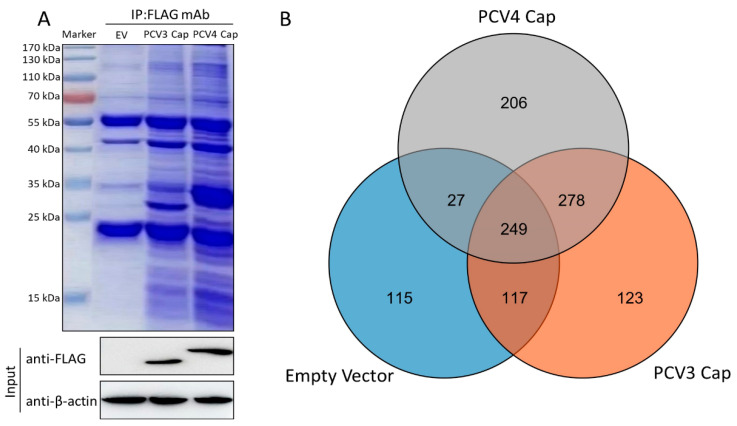
Characterization of PCV3 or PCV4 Cap-interacting cellular proteins. (**A**) Empty vector, PCV3, or PCV4 Cap-transfected PK-15 cells were harvested at 48 hpt, and a co-immunoprecipitation assay was performed using anti-FLAG beads. PCV3 or PCV4 Cap-interacting host proteins were eluted and analyzed via SDS-PAGE followed by Coomassie blue staining. Lane 1, protein molecular weight ladder; lane 2, empty vector-transfected; lane 3, PCV3 Cap-transfected; lane 4, PCV4 Cap-transfected. (**B**) Venn diagram of the identified protein candidates interacting with PCV3 or PCV4 Cap from the empty vector-transfected, PCV3 Cap-transfected, and PCV4 Cap-transfected cells, respectively. Blue, orange, and gray colors indicate proteins from the empty vector-transfected, PCV3 Cap-transfected, and PCV4 Cap-transfected cells, respectively. Common proteins within the data sets are indicated in the colored intersections. Proteins were represented as their respective NCBI gene names (Supplementary Table S1).

To map host protein interactions with PCV3 or PCV4 Cap in transfected PK-15 cells, we conducted Co-IP assays coupled with LC-MS/MS with or without PCV3 or PCV4 Cap expression. Whole-cell lysates were co-immunoprecipitated with anti-FLAG beads at 48 h post-transfection (hpt), and Coomassie blue staining was performed to visualize host proteins binding to PCV3 or PCV4 Cap (Figure 1A). As a negative control, empty vector-transfected PK-15 cell lysates were adopted to remove non-specifically bound proteins. Differential bands were visualized compared to the negative control. LC-MS was used to elucidate the cellular proteins bound to PCV3 or PCV4 Cap. In total, 401 PCV3 Cap- and 484 PCV4 Cap-specifically expressed and bound cellular protein candidates were characterized in transfected PK-15 cells (Figure 1B and Supplementary Table S1). Among them were 123 putative PCV3 Cap- and 206 putative PCV4 Cap-specific interacting proteins and 278 common host proteins, which might result in differences in porcine circovirus replication and pathogenesis. Hence, these proteins were subjected to further analyses (Figure 1B and Supplementary Tables S2 and S3).

### 3.2. Construction of a Protein–Protein Interaction Network

**Figure 2:**
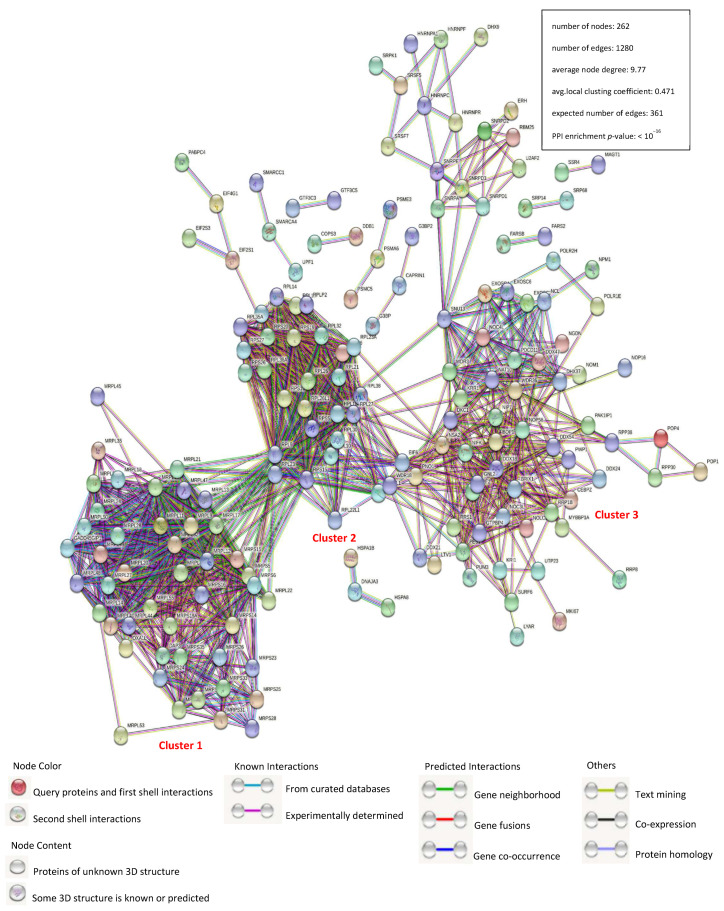
Construction and analysis of the protein-protein interaction network using the STRING database. Each edge color indicates a different method of protein–protein interaction prediction, as indicated below the figure. A map of the interaction of 278 identical bound host proteins shared amongst PCV3 and PCV4 Caps with the other proteins in our data was constructed and plotted using the network analyzer tool of the Cytoscape software, version 3.7.1. The corresponding symbols indicating the different protein classes are shown. Proteins are represented by their respective NCBI gene names (Supplementary Table S2).

Verifying protein–protein interactions (PPIs) is a critical aspect of molecular biology because of the incontestable role of cellular factors. Herein, we plotted an interaction network of identical PCV3 and PCV4 Cap-binding cellular host proteins with the STRING database and used it for network structure and functional analyses (Figure 2 and Supplementary Table S2). Together with the verified protein interactions, those acquired from gene fusion, co-expression, homology, and text mining were used for the construction of network as well. The host proteins in the interaction network were predominantly divided into ribosome biogenesis modulation, cellular amide metabolism, and cytoskeletal reshape. There are three large and distinct clusters. Cluster 1 proteins are mainly mitochondrial ribosomal proteins (MRPs) and classified into two main categories: MRPL, components of the large subunit; MRPS, components of the small subunit. Cluster 2 proteins are mainly ribosomal proteins (RPs) and are classified into two main categories: RPL, components of the large subunit; RPS, components of the small subunit. Cluster 3 proteins are mainly DEAD-box RNA helicases. The number of edges for the network (1280) was notably higher than the expected number (361) for a constant number of nodes (262), indicating that there were more interactions than expected and that the data showed more interactions than expected for a random set of proteins. The results demonstrated that the proteins were partially divided into several roles, mostly in replication or transcription.

### 3.3. Gene Ontology Annotation

**Figure 3:**
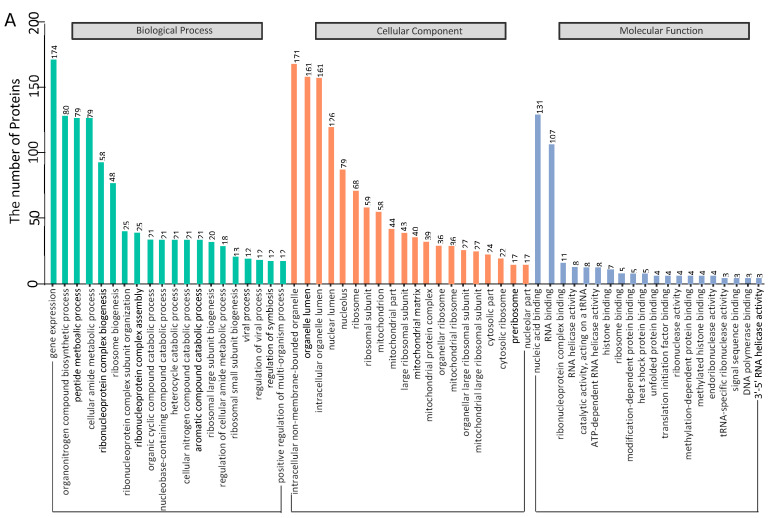

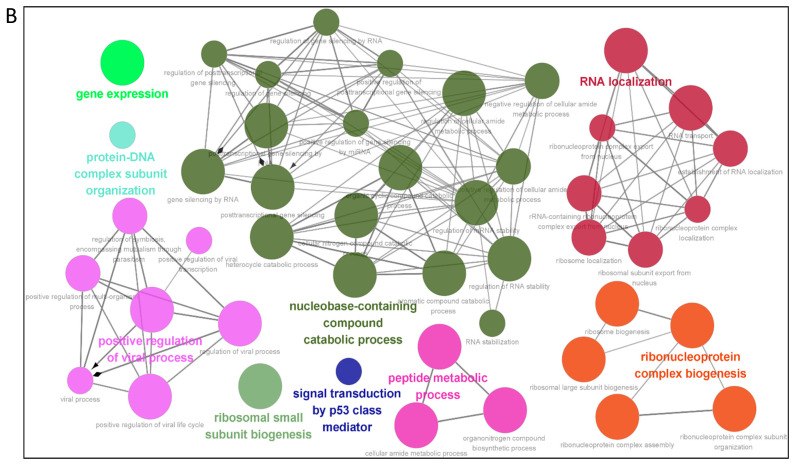
Gene ontology analysis of the identified PCV3 and PCV4 Cap-host interactome. (**A**,**B**) Representative overrepresented GO terms of protein clusters and the GO distribution of all proteins in PCV3 and PCV4 Cap-transfected cells versus empty vector-transfected cells were classified into three categories using the GOclue plugin in Cytoscape software, version 3.7.1. Shown are the significantly enriched terms based on biological process (BP), molecular function (MF), and cellular component (CC) with *p*-values < 0.05. The Roman numerals represent the detailed GO terms, as shown in Supplementary Table S4.

To identify cellular pathways in the PCV3 or PCV4 Cap-host protein interaction network, we conducted gene ontology annotation of the proteins common to PCV3 or PCV4 Cap-transfected PK-15 cells (Supplementary Table S2) to forecast their molecular functions. GO annotation was performed for the following three categories: biological processes, molecular functions, and cellular components. Many biological processes, such as gene expression, organonitrogen compound biosynthesis, peptide metabolism, cellular amide metabolism, and ribonucleoprotein complex biogenesis, were enriched. In addition, nucleic acid binding, RNA binding, ribonucleoprotein complex binding, and ATP-dependent RNA helicase activity were enriched within the category of molecular function, while intracellular non-membrane-bounded organelles, intracellular organelle lumen, and nuclear lumen were enriched under the category of cellular components (Figure 3A,B and Supplementary Table S4). In summary, GO annotation of common proteins suggested that PCV3 or PCV4 Cap might disturb some processes such as ribosome biogenesis, nucleic acid binding, and ATP-dependent RNA helicase activity. 

### 3.4. KEGG Pathway Enrichment

**Figure 4:**
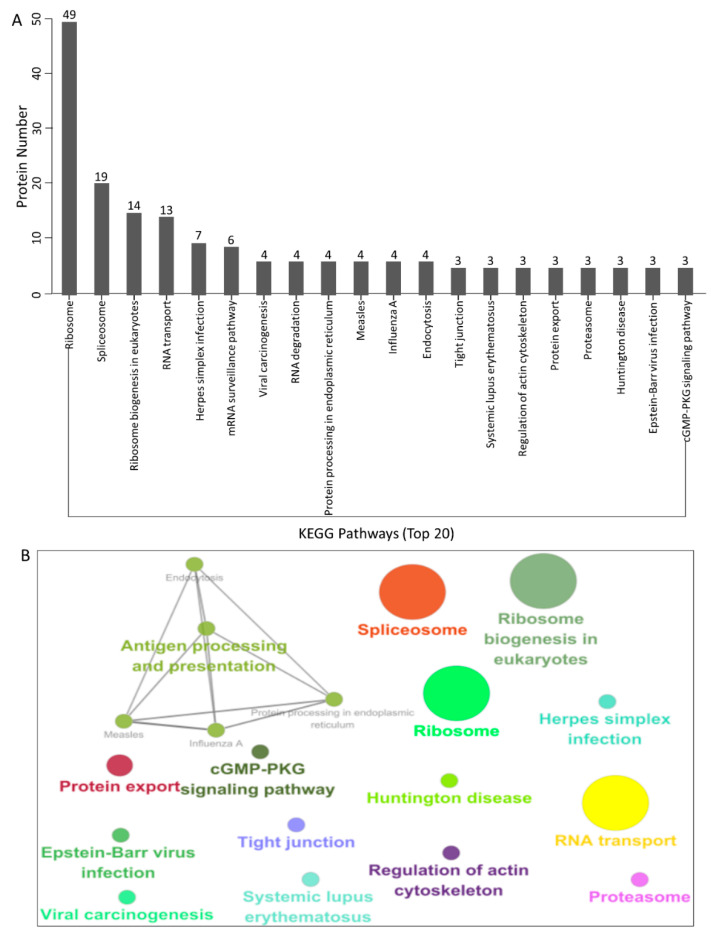
KEGG pathway enrichment analysis. (**A**,**B**) Graphs showing the enriched pathways targeted by the PCV3 and PCV4 Cap-interacting proteins, as analyzed via KEGG functional annotation (Supplementary Table S5) using the GOclue plugin in Cytoscape software, version 3.7.1.

To further establish the host signal transduction pathways associated with Cap-interacting host proteins targeted by PCV3 and PCV4 (Supplementary Table S2), we performed KEGG pathway enrichment, and the top 20 enriched pathways with the highest representation of each term are obtained. Remarkably, most of the potential proteins were involved in the ribosomes, spliceosomes, RNA transport, Herpes simplex infection, mRNA surveillance, RNA degradation, and the cGMP-PKG signaling pathway. KEGG enrichment indicated that pathways involving ribosomes, RNA transport and degradation, and influenza A infection were preferentially enriched (Figure 4A,B and Supplementary Table S5). In addition, KEGG enrichment demonstrated that these proteins may play a crucial role in the regulation of distinct processes, such as endocytosis, Huntington’s disease, and Epstein–Barr virus infection.

### 3.5. Validation of the Interactions between Host Proteins and PCV3 or PCV4 Capsid Proteins

**Figure 5:**
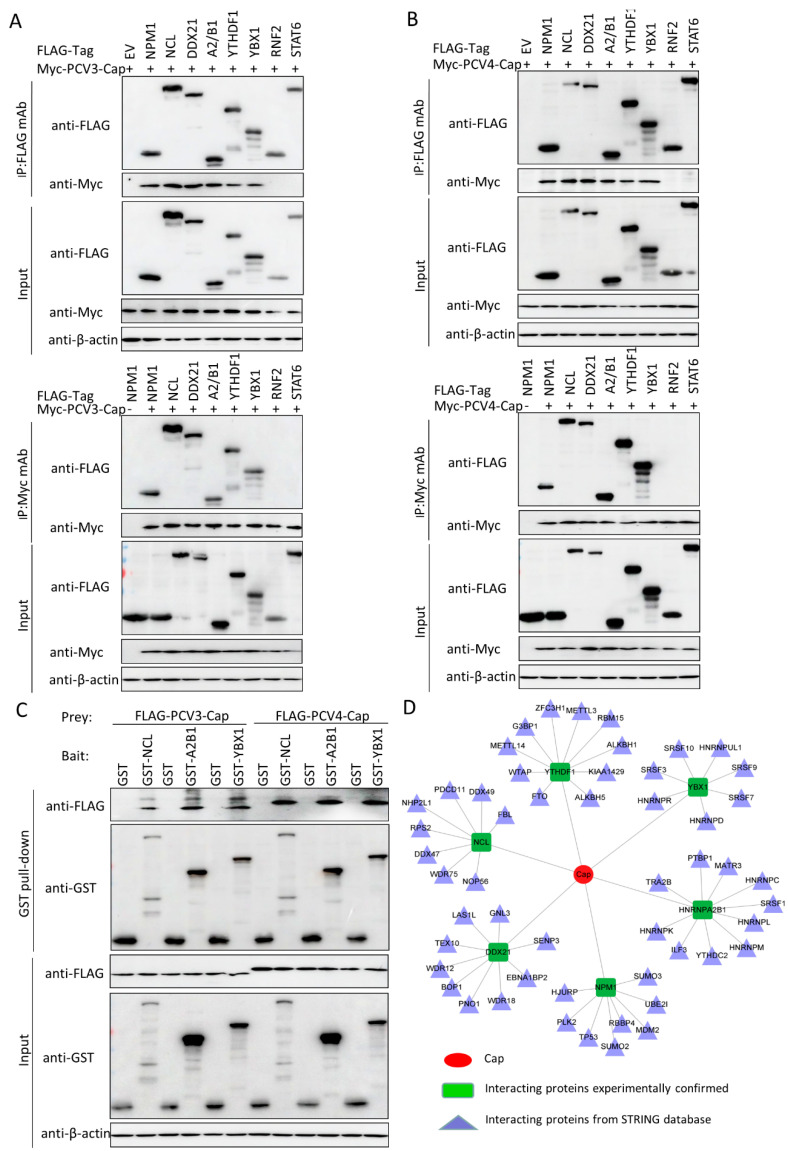
Validation of Cap-host protein interactions. (**A**,**B**) HEK293T cells were co-transfected with plasmids expressing FLAG-NPM1, FLAG-NCL, FLAG-DDX21, FLAG-hnRNPA2/B1, FLAG-YTHDF1, FLAG-YBX1, FLAG-RNF2, or FLAG-STAT6 and plasmids expressing Myc-PCV3-Cap (**A**), or Myc-PCV4-Cap (**B**), respectively. Among them, Myc-PCV3-Cap (**A**), Myc-PCV4-Cap (**B**), or FLAG-NPM1 (**A**,**B**) co-transfected with an empty vector served as negative controls, while Myc-PCV3-Cap (**A**) or Myc-PCV4-Cap (**B**) co-transfected with FLAG-NPM1 served as positive controls. Cell lysates were immunoprecipitated with FLAG beads or anti-Myc mAbs, separated via SDS-PAGE, and then subject to Western blotting with the corresponding primary and secondary antibodies. β-actin served as the internal loading control. (**C**) Whole-cell lysates of PCV3 or PCV4 Cap were separately added to the GST, GST-NCL, GST-hnRNPA2/B1, or GST-YBX1 proteins; subjected to GST pull-down assays; and then immunoblotted with the corresponding primary and secondary antibodies. (**D**) The PCV3 and PCV4 Cap-host interaction network. The interaction map of PCV3 and PCV4-Cap and the corresponding host proteins was constructed using Cytoscape software, version 3.7.1. Proteins were classified based on their protein class. The corresponding symbols indicating different protein classes are mentioned in the figure (Supplementary Table S6).

To further verify protein interactions from mass spectrometry, we performed in vitro Co-IP assays. Eight host proteins from PCV3 or PCV4 Cap-transfected samples were randomly selected to validate mass spectrometry data. HEK293T cells were co-transfected with Myc-PCV3-Cap or Myc-PCV4-Cap and empty vectors; FLAG-NPM1, NCL, DDX21, hnRNPA2/B1, YTHDF1, YBX1, RNF2, or STAT6 expression constructs; and then subjected to immunoprecipitation with FLAG beads or anti-Myc purified monoclonal antibody (mAb). The results indicated that PCV3 or PCV4 Cap bound specifically to NPM1, NCL, DDX21, hnRNPA2/B1, YTHDF1, and YBX1, while no signal was observed with the empty vector, RNF2, and STAT6 constructs, although they exhibited distinct binding capacities (Figure 5A,B). To confirm whether Cap interacts directly with six cellular proteins, we conducted glutathione S-transferase (GST) pull-down experiments. Lysates of FLAG-PCV3-Cap-, FLAG-PCV4-Cap-, GST, GST-NCL-, GST-hnRNPA2/B1-, and GST-YBX1-transfected cells were subjected to GST pull-down and immunoblotting assays, respectively. As shown in Figure 5C, GST-NCL, GST-hnRNPA2/B1, and GST-YBX1 pulled down FLAG-PCV3-Cap or FLAG-PCV4-Cap. In summary, the results demonstrated that PCV3 or PCV4 Cap binds directly to NCL, hnRNPA2/B1, and YBX1. Hence, the results acquired from Co-IP and GST pull-down assays verified the data from the LC-MS-based proteomic analyses. Next, we plotted another interaction network of the experimentally validated cellular protein-PCV3 or PCV4 Cap interactions and the host partners of the PCV3 and PCV4 Cap-binding cellular proteins in silico with Cytoscape software version 3.7.1 (Figure 5D and Supplementary Table S6), which may be useful for studying the underlying role of PCV3 or PCV4 Cap in the viral lifecycle.

## 4. Discussion

Virus–host interactions interplay between pathogenicity and immunity, leading to either the activation of the host immune defense system to eliminate the virus or the utilization of cellular immune mechanisms to promote virus proliferation. Virus–host interaction plays an important role in the viral lifecycle, initiating the cascade between pathogenesis and host immunity. The circovirus Cap protein is a key controller of virus replication along with the Rep protein and is required for viral propagation [24,33]. Thus, it is speculated that the Cap and Rep proteins of PCV3 or PCV4 might form a replicase complex by binding to many cellular proteins and that this multiprotein complex might play a critical role in virus uncoating, assembly, nuclear entry, and egress. However, it remains unclear whether the PCV3 or PCV4 Cap protein subverts or utilizes the host machinery for virus replication. In addition, the domains of the Cap protein essential for the interaction with cellular proteins have been identified.

Previous research has reported the interactions of PCV1 and PCV2 Cap with cellular proteins [24,25,33,34,35,36,37,38]. In this study, we characterized 401 and 484 putative host proteins binding to PCV3 or PCV4 Cap, respectively, via Co-IP and LC-MS (Figure 1). Even though the interacting cellular proteins cannot exhibit fold changes in expression levels under PCV3 or PCV4 infection, they can also be used for further study. We previously identified 222 putative PCV2 Cap-binding cellular proteins [25]. In the present study, 278 identical interacting cellular proteins and 123 putative PCV3 Cap- or 206 putative PCV4 Cap-unique binding host proteins were identified, which might play crucial roles in porcine circovirus pathogenesis, highlighting the characteristic differences between PCV3 and PCV4. For example, the Rho or Rab family GTPases Cdc42 and Rab35, which specifically interact with PCV3 Cap, are key moderators of cellular actin dynamics. The specific Cdc42/Rab35-inhibiting agents provide unprecedented ability to investigate their roles in various signaling pathways [42,43]. Cellular protein high-mobility group box 1 (HMGB1), which binds to PCV4 Cap, is a multifunctional protein with various roles in different cellular compartments. It acts as a chromosome guardian and DNA chaperone involved in DNA replication, gene transcription, DNA repair, nucleosome stability, telomere homeostasis, and PCV2 replication [44,45,46]. Since the amino acid identity between PCV3 and PCV4 Caps is below 24.5%, we chose to explore only the host proteins shared amongst PCV3 Cap and PCV4 Caps for better evaluating the similar cellular protein functions and pathways that are related to both PCV3 and PCV4 Caps. However, we will conduct the GO and KEGG analyses of the unique host cellular proteins to make it more objective, comprehensive, and informative in the future. Thus, we recommend that future studies utilize all information to select potential proteins essential for PCV infection.

After that, a PPI network was constructed (Figure 2). Gene ontology (GO) and the Kyoto Encyclopedia of Genes and Genomes (KEGG) pathway analyses showed that the binding host proteins participate in diverse biological processes, such as gene expression, cellular amide metabolic process, nucleic acid binding, ribonucleoprotein complex binding, and ATP-dependent RNA helicase activity (Figure 3 and Figure 4). We also verified the interactions of PCV3 or PCV4 Cap with potential cellular proteins, such as NPM1, NCL, DDX21, hnRNPA2/B1, YTHDF1, and YBX1 in vitro using Co-IP and GST pull-down experiments (Figure 5). To date, our current study was the first study that used modern proteomic tools such as LC-MS to identify the host proteins interacting with PCV3 or PCV4 Cap. Bioinformatics analyses of available datasets will be helpful for understanding the roles of cellular proteins in biological pathways better. Advances in tools for data acquisition, processing, integration, and computation could provide more rapid and precise strategies for the development of therapies for infectious diseases [47,48,49]. Therefore, we speculated that cellular proteins binding to PCV3 or PCV4 Cap might constitute a differential replicase complex that plays a significant role in the replication and pathogenesis of PCV3 or PCV4.

Several biological processes, such as ribosome biogenesis, nucleic acid binding, and ATP-dependent RNA helicase activity, are important during PCV3 or PCV4 infection and require special attention in future studies. In this study, although we have characterized some proteins associated with these pathways, their exact roles are still unclear; thus, further studies are essential. An apparent feature of virus–host interactions is their ability to manipulate the innate immune response to favor virus multiplication. It is hypothesized that PCV3 or PCV4 might manipulate the innate immune response pathways. To support this hypothesis, our GO annotation and KEGG enrichment analyses demonstrated that proteins related to these pathways were enriched (Figure 3 and Figure 4). In addition, our results indicated the enrichment of the spliceosome pathway. Different heterogeneous nuclear ribonucleoproteins such as hnRNPA2B1, hnRNPC, and hnRNPU were confirmed to bind to PCV3 or PCV4 Cap (Figure 5). Spliceosomal complex proteins are required to generate stable RNA structures, and ribonucleoproteins play a role in RNA stability [50]. Previous reports have demonstrated that proteins hnRNPA2B1 and hnRNPC are associated with influenza A virus (IAV) [51,52], human immunodeficiency virus type 1 (HIV-1) [53], herpes simplex virus 1 (HSV-1) [54,55], hepatitis delta virus (HDV) [56], dengue virus (DENV) [57], and Japanese encephalitis virus (JEV) infections [58], while hnRNPU acts as a nuclear sensor for viral RNA [59].

In the current study, Cap-cellular protein interactions were identified for the first time in PCV3 or PCV4 Cap-transfected PK-15 cells. A protein–protein interaction network was plotted, and the potential functions of the characterized cellular proteins were predicted via GO and KEGG enrichment analyses. Six of the eight randomly selected proteins could interact with PCV3 or PCV4 Cap, as observed from the results of the Co-IP and GST pull-down assays. The interactions of PCV3 or PCV4 Cap with cellular proteins and the interpretation of the virus–host interaction network would be useful to better understand the putative mechanisms through which PCV3 or PCV4 exert their pathogenic effects. Moreover, the results also suggest that the replication mechanism and pathogenesis of PCV3 and PCV4 are complex phenomena and require further research. Increased knowledge about the cellular proteins targeted and the pathways perturbed by PCV3 or PCV4 Cap might contribute to the extensive knowledge of virus–host interactions and provide new insights for identifying novel targets. Ultimately, this information would help design better therapeutic strategies against PCVAD.

## Figures and Tables

**Table 1:** List of primers used for cloning in the study.

Gene Product	Sense Primer (5′ to 3′)	Antisense Primer (5′ to 3′)
*PCV3 Cap*	ATGAGACACAGAGCTATATTC	TTAGAGAACGGACTTGTAACGAATC
*PCV4 Cap*	ATGCCAATCAGATCTAGGTACA	TTATCCCTGTTTGGGGTAGTTAACA
*NPM1*	ATGGAAGATTCGATGGATAT	TTAAAGAGACTTCCTCCACT
*NCL*	ATGGTAAAGCTCGCAAAGGCC	CTATTCAAACTTGGTCTTCTTTCCT
*DDX21*	ATGCCGGGGAAACTTCGTAGT	TTACTGTCCAAACGCTTTGCT
*hnRNPA2/B1*	ATGGAGAAAACTTTAGAAACTGT	TCAATATCGGCTTCTCCCTCCAT
*YTHDF1*	ATGTCGGCCACCAGCGTGGACC	TTATTGTTTGTTTCGATTCTGCCGT
*YBX1*	ATGAGCAGCGAGGCCGAGA	TTACTCAGCCCCGCCCTGCTCA
*RNF2*	ATGTCTCAGGCTGTGCAGACAAAT	TCATTTGTGCTCCTTTGTGGGT
*STAT6*	ATGTCTCTGTGGGGTCTGGTCTCCAAAAT	TCACCAACTGGGGTTAGCCCTTAGGT

## Data Availability

All datasets generated for this study are included in the article/Supplementary Materials.

## Supplementary Materials

The following supporting information can be downloaded at: www.mdpi.com/article/10.3390/v14050939/s1, Table S1: The PCV3 or PCV4 Cap-interacting cellular proteins in the PCV3 or PCV4 Cap or empty vector transfected PK-15 cells, respectively; Table S2: The common PCV3 or PCV4 Cap binding host proteins (278); Table S3: The unique PCV3 Cap (123) or PCV4 Cap binding cellular proteins (206); Table S4: The GO annotation analyses of PCV3 or PCV4 Cap-interacting cellular proteins; Table S5: The KEGG enrichment analyses of PCV3 or PCV4 Cap-interacting host proteins; Table S6: The interactions network of the confirmed host protein-PCV3 or PCV4 Cap interaction and the cellular partners of the Cap-interacting host proteins.

## References

[1] Breitbart M., Delwart E., Rosario K., Segales J., Varsani A. (2017). ICTV Report Consortium ICTV Virus Taxonomy Profile: Circoviridae. J. Gen. Virol..

[2] Zhang H.H., Hu W.Q., Li J.Y., Liu T.N., Zhou J.Y., Opriessnig T., Xiao C.T. (2019). Novel circovirus species identified in farmed pigs designated as Porcine circovirus 4, Hunan province, China. Transbound. Emerg. Dis..

[3] Oh T., Chae C. (2020). First isolation and genetic characterization of porcine circovirus type 3 using primary porcine kidney cells. Vet. Microbiol..

[4] Opriessnig T., Karuppannan A.K., Castro A.M., Xiao C.-T. (2020). Porcine circoviruses: Current status, knowledge gaps and challenges. Virus Res..

[5] Tischer I., Gelderblom H.R., Vettermann W., Koch M.A. (1982). A very small porcine virus with circular single-stranded DNA. Nature.

[6] Allan G.M., McNeilly F., Kennedy S., Daft B., Clarke E.G., Ellis J.A., Haines D.M., Meehan B.M., Adair B.M. (1998). Isolation of Porcine Circovirus-like Viruses from Pigs with a Wasting Disease in the USA and Europe. J. Vet. Diagn. Investig..

[7] Phan T.G., Giannitti F., Rossow S., Marthaler D., Knutson T.P., Li L., Deng X., Resende T., Vannucci F., Delwart E. (2016). Detection of a novel circovirus PCV3 in pigs with cardiac and multi-systemic inflammation. Virol. J..

[8] Palinski R., Piñeyro P., Shang P., Yuan F., Guo R., Fang Y., Byers E., Hause B.M. (2017). A Novel Porcine Circovirus Distantly Related to Known Circoviruses Is Associated with Porcine Dermatitis and Nephropathy Syndrome and Reproductive Failure. J. Virol..

[9] Jiang H., Wang D., Wang J., Zhu S., She R., Ren X., Tian J., Quan R., Hou L., Li Z. (2019). Induction of Porcine Dermatitis and Nephropathy Syndrome in Piglets by Infection with Porcine Circovirus Type 3. J. Virol..

[10] Sun W., Du Q., Han Z., Bi J., Lan T., Wang W., Zheng M. (2020). Detection and genetic characterization of porcine circovirus 4 (PCV4) in Guangxi, China. Gene.

[11] Chen N., Xiao Y., Li X., Li S., Xie N., Yan X., Li X., Zhu J. (2020). Development and application of a quadruplex real-time PCR assay for differential detection of porcine circoviruses (PCV1 to PCV4) in Jiangsu province of China from 2016 to 2020. Transbound. Emerg. Dis..

[12] Ha Z., Yu C., Xie C., Wang G., Zhang Y., Hao P., Li J., Li Z., Li Y., Rong F. (2021). Retrospective surveillance of porcine circovirus 4 in pigs in Inner Mongolia, China, from 2016 to 2018. Arch. Virol..

[13] Tian R., Zhao Y., Cui J., Zheng H., Xu T., Hou C., Wang Z., Li X., Zheng L., Chen H. (2020). Molecular detection and phylogenetic analysis of *Porcine circovirus* 4 in Henan and Shanxi Provinces of China. Transbound. Emerg. Dis..

[14] Nguyen V., Do H., Huynh T., Park Y., Park B., Chung H. (2021). Molecular-based detection, genetic characterization and phylogenetic analysis of porcine circovirus 4 from Korean domestic swine farms. Transbound. Emerg. Dis..

[15] Hamel A.L., Lin L.L., Nayar G.P.S. (1998). Nucleotide Sequence of Porcine Circovirus Associated with Postweaning Multisystemic Wasting Syndrome in Pigs. J. Virol..

[16] Cheung A.K. (2003). Transcriptional analysis of porcine circovirus type 2. Virology.

[17] Zhou J.-Y., Chen Q.-X., Ye J.-X., Shen H.-G., Chen T.-F., Shang S.-B. (2006). Serological Investigation and Genomic Characterization of PCV2 Isolates from Different Geographic Regions of Zhejiang Province in China. Vet. Res. Commun..

[18] Mankertz A., Mankertz J., Wolf K., Buhk H.J. (1998). Identification of a protein essential for replication of porcine circovirus. J. Gen. Virol..

[19] Mankertz A., Hillenbrand B. (2001). Replication of porcine circovirus type 1 requires two proteins encoded by the viral rep gene. Virology.

[20] Cheung A.K. (2006). Rolling-circle replication of an animal circovirus genome in a theta-replicating bacterial plasmid in Escherichia coli. J. Virol..

[21] Nawagitgul P., Morozov I., Bolin S.R., Harms P.A., Sorden S.D., Paul P.S. (2000). Open reading frame 2 of porcine circovirus type 2 encodes a major capsid protein. J. Gen. Virol..

[22] Blanchard P., Mahe D., Cariolet R., Keranflec’h A., Baudouard M.A., Cordioli P., Albina E., Jestin A. (2003). Protection of swine against post-weaning multisystemic wasting syndrome (PMWS) by porcine circovirus type 2 (PCV2) proteins. Vaccine.

[23] Fenaux M., Opriessnig T., Halbur P.G., Elvinger F., Meng X.J. (2004). Two amino acid mutations in the capsid protein of type 2 porcine circovirus (PCV2) enhanced PCV2 replication in vitro and attenuated the virus in vivo. J. Virol..

[24] Timmusk S., Fossum C., Berg M. (2006). Porcine circovirus type 2 replicase binds the capsid protein and an intermediate filament-like protein. J. Gen. Virol..

[25] Zhou J., Li H., Yu T., Li J., Dong W., Ojha N.K., Jin Y., Gu J., Zhou J. (2020). Protein interactions network of porcine circovirus Type 2 capsid with host proteins. Front. Microbiol..

[26] Liu J., Chen I., Kwang J. (2005). Characterization of a previously unidentified viral protein in porcine circovirus type 2-infected cells and its role in virus-induced apoptosis. J. Virol..

[27] He J., Cao J., Zhou N., Jin Y., Wu J., Zhou J. (2013). Identification and functional analysis of the novel ORF4 protein encoded by porcine circovirus type 2. J. Virol..

[28] Lv Q., Guo K., Xu H., Wang T., Zhang Y. (2015). Identification of putative ORF5 protein of porcine circovirus type 2 and functional analysis of GFP-fused ORF5 protein. PLoS ONE.

[29] Li D., Wang J., Xu S., Cai S., Ao C., Fang L., Xiao S., Chen H., Jiang Y. (2017). Identification and functional analysis of the novel ORF6 protein of porcine circovirus type 2 in vitro. Vet. Res. Commun..

[30] Heath L., Williamson A.L., Rybicki E.P. (2006). The capsid protein of beak and feather disease virus binds to the viral DNA and is responsible for transporting the replication-associated protein into the nucleus. J. Virol..

[31] Asensio N.C., Giner E.M., De Groot N.S., Burgas M.T. (2017). Centrality in the host–pathogen interactome is associated with pathogen fitness during infection. Nat. Commun..

[32] Fermin G., Tennant P. (2018). Host-Virus Interactions: Battles between Viruses and Their Hosts.

[33] Finsterbusch T., Steinfeldt T., Doberstein K., Rodner C., Mankertz A. (2009). Interaction of the replication proteins and the capsid protein of porcine circovirus type 1 and 2 with host proteins. Virology.

[34] Zhang X., Zhou J., Wu Y., Zheng X., Ma G., Wang Z., Jin Y., He J., Yan Y. (2009). Differential proteome analysis of host cells infected with porcine circovirus type 2. J. Proteome Res..

[35] Liu J., Bai J., Zhang L., Jiang Z., Wang X., Li Y., Jiang P. (2013). Hsp70 positively regulates porcine circovirus type 2 replication in vitro. Virology.

[36] Wang Z.J., Xu C.M., Song Z.B., Wang M., Liu Q.Y., Jiang P., Li Y.F., Bai J., Wang X.W. (2018). Vimentin modulates infectious porcine circovirus type 2 in PK-15 cells. Virus Res..

[37] Cao J., Lin C., Wang H., Wang L., Zhou N., Jin Y., Liao M., Zhou J. (2015). Circovirus transport proceeds via direct interaction of the cytoplasmic dynein IC1 subunit with the viral capsid protein. J. Virol..

[38] Wang T., Du Q., Niu Y., Zhang X., Wang Z., Wu X., Yang X., Zhao X., Liu S.-L., Tong D. (2019). Cellular p32 Is a Critical Regulator of Porcine Circovirus Type 2 Nuclear Egress. J. Virol..

[39] Zhou J.W., Dai Y.D., Lin C., Zhang Y., Feng Z.X., Dong W.R., Jin Y.L., Yan Y., Zhou J.Y., Gu J.Y. (2020). Nucleolar protein NPM1 is essential for circovirus replication by binding to viral capsid. Virulence.

[40] Zhou J., Li J., Li H., Zhang Y., Dong W., Jin Y., Yan Y., Gu J., Zhou J. (2021). The serine-48 residue of nucleolar phosphoprotein nucleophosmin-1 plays critical role in subcellular localization and interaction with porcine circovirus type 3 capsid protein. Veter Res..

[41] Zhou J., Qiu Y., Zhu N., Zhou L., Dai B., Feng X., Hou L., Liu J. (2021). The Nucleolar Localization Signal of Porcine Circovirus Type 4 Capsid Protein Is Essential for Interaction With Serine-48 Residue of Nucleolar Phosphoprotein Nucleophosmin-1. Front. Microbiol..

[42] Swaine T., Dittmar M.T. (2015). CDC42 use in viral cell entry processes by RNA viruses. Viruses.

[43] Klinkert K., Echard A. (2016). Rab35 GTPase: A central regulator of phosphoinositides and F-actin in endocytic recycling and beyond. Traffic.

[44] Kang R., Chen R., Zhang Q., Hou W., Wu S., Cao L., Huang J., Yu Y., Fan X.-G., Yan Z. (2014). HMGB1 in health and disease. Mol. Asp. Med..

[45] Sun R., Sun S., Zhang Y., Zhou Y., Shan Y., Li X., Fang W. (2020). PCV2 Induces Reactive Oxygen Species to Promote Nucleocytoplasmic Translocation of the Viral DNA Binding Protein HMGB1 To Enhance Its Replication. J. Virol..

[46] Sun R., Deng Z., Han X., Zhang Y., Zhou Y., Shan Y., Fang W., Li X. (2021). Porcine Circovirus 2 Manipulates the PERK-ERO1α Axis of the Endoplasmic Reticulum To Favor Its Replication by Derepressing Viral DNA from HMGB1 Sequestration within Nuclei. J. Virol..

[47] Aderem A., Adkins J.N., Ansong C., Galagan J., Kaiser S., Korth M.J., Law G.L., McDermott J.G., Proll S.C., Rosenberger C. (2011). A Systems Biology Approach to Infectious Disease Research: Innovating the Pathogen-Host Research Paradigm. mBio.

[48] Peng X., Chan E.Y., Li Y., Diamond D.L., Korth M.J., Katze M.G. (2009). Virus–host interactions: From systems biology to translational research. Curr. Opin. Microbiol..

[49] Xue Q., Miller-Jensen K. (2012). Systems biology of virus-host signaling network interactions. BMB Rep..

[50] Will C.L., Lührmann R. (2010). Spliceosome Structure and Function. Cold Spring Harb. Perspect. Biol..

[51] Chang C.-K., Chen C.-J., Wu C.-C., Chen S.-W., Shih S.-R., Kuo R.-L. (2017). Cellular hnRNP A2/B1 interacts with the NP of influenza A virus and impacts viral replication. PLoS ONE.

[52] Wang Y.M., Zhou J.H., Du Y.C. (2014). hnRNP A2/B1 interacts with influenza A viral protein NS1 and inhibits virus replication potentially through suppressing NS1 RNA/protein levels and NS1 mRNA nuclear export. Virology.

[53] Gordon H., Ajamian L., Valiente-Echeverria F., Levesque K., Rigby W.F., Mouland A.J. (2013). Depletion of hnRNP A2/B1 overrides the nuclear retention of the HIV-1 genomic RNA. RNA Biol..

[54] Zhou X., Wang L., Zou W., Chen X., Roizman B., Zhou G.G. (2020). hnRNPA2B1 Associated with Recruitment of RNA into Exosomes Plays a Key Role in Herpes Simplex Virus 1 Release from Infected Cells. J. Virol..

[55] Wang L., Wen M., Cao X. (2019). Nuclear hnRNPA2B1 initiates and amplifies the innate immune response to DNA viruses. Science.

[56] Casaca A., Fardilha M., Silva E.D.C.E., Cunha C. (2011). The heterogeneous ribonuclear protein C interacts with the hepatitis delta virus small antigen. Virol. J..

[57] Dechtawewat T., Songprakhon P., Limjindaporn T., Puttikhunt C., Kasinrerk W., Saitornuang S., Yenchitsomanus P.-T., Noisakran S. (2015). Role of human heterogeneous nuclear ribonucleoprotein C1/C2 in dengue virus replication. Virol. J..

[58] Mukherjee S., Singh N., Sengupta N., Fatima M., Seth P., Mahadevan A., Shankar S.K., Bhattacharyya A., Basu A. (2017). Japanese encephalitis virus induces human neural stem/progenitor cell death by elevating GRP78, PHB and hnRNPC through ER stress. Cell Death Dis..

[59] Cao L., Liu S., Li Y., Yang G., Luo Y., Li S., Du H., Zhao Y., Wang D., Chen J. (2019). The Nuclear Matrix Protein SAFA Surveils Viral RNA and Facilitates Immunity by Activating Antiviral Enhancers and Super-enhancers. Cell Host Microbe.

